# Hsp47 Inhibitor Col003 Attenuates Collagen-Induced Platelet Activation and Cerebral Ischemic–Reperfusion Injury in Rats

**DOI:** 10.3389/fphar.2021.792263

**Published:** 2022-01-10

**Authors:** Shuang Wu, Chengwei Liang, Xiaoyun Xie, Haiping Huang, Jinfeng Fu, Cilan Wang, Zhiheng Su, Youqiong Wang, Xiang Qu, Jinpin Li, Jingli Liu

**Affiliations:** ^1^ Department of Neurology, The First Affiliated Hospital of Guangxi Medical University, Nanning, China; ^2^ College of Pharmacy, Guangxi Medical University, Nanning, China

**Keywords:** ischemic stroke, antiplatelet, Col003, HSP47, GPVI

## Abstract

Ischemic stroke is a major type of stroke worldwide currently without effective treatment, although antiplatelet therapy is an existing option for it. In previous studies, heat shock protein 47 (Hsp47) was found to be expressed on the surface of human and mice platelets and to strengthen the interaction between platelets and collagen. In recent years, Col003 was discovered to inhibit the interaction of Hsp47 with collagen. We evaluated whether the Hsp47 inhibitor Col003 is a promising therapeutic agent for ischemic stroke. Here, we first verified that Hsp47 is also expressed on the surface of rat platelets, and its inhibitor Col003 significantly inhibited thrombus formation in the FeCl_3_-induced rat carotid arterial thrombus model. Both Col003 and clopidogrel did not alter the bleeding time or coagulation parameters, while aspirin increased the tail-bleeding time (*p* < 0.05). The low cytotoxicity level of Col003 to rat platelets and human liver cells was similar to those of aspirin and clopidogrel. Col003 inhibited collagen-induced platelet aggregation, adhesion, [Ca^2+^]_i_ mobilization, P-selectin expression, reactive oxygen species production and the downstream signal pathway of collagen receptors. The results of the middle cerebral artery occlusion model indicated that Col003 has a protective effect against cerebral ischemic–reperfusion injury in rats. The Hsp47 inhibitor Col003 exerted antiplatelet effect and protective effect against brain damage induced by ischemic stroke through the inhibition of glycoprotein VI (GPVI)and mitogen-activated protein kinase (MAPK) signaling events, which might yield a new antiplatelet agent and strategy to treat ischemic stroke.

## Introduction

Ischemic stroke is a highly disabling disease with high morbidity and mortality rates worldwide (Phipps and Cronin, 2020) that is caused by a transient or permanent occlusion of the cerebral blood flow, particularly in the MCA. Though an important conventional pharmacotherapy for ischemic brain injuries, antiplatelet therapy is associated with drug resistance and a high risk of bleeding (Diener and Hankey, 2020). The safety and efficacy of existing treatments of antiplatelet therapy are limited (Kamran et al., 2021), and novel antiplatelet agents are required to reduce pathological thrombosis and bleeding.

At sites of vascular injury, platelets can adhere to the subendothelium by the interaction between glycoprotein (GP) Ib receptor and von Willebrand factor (Wang et al., 2021). This tethering of platelets further facilitates their firm binding to subendothelial collagen through the engagement of collagen receptors GPVI and α2β1, resulting in an intracellular signaling cascade that initiates platelet activation (Kahner et al., 2007). Given that activated platelets have a dual role in hemostasis and thrombosis, current and future antiplatelet therapies must place great emphasis on preserving hemostasis while inhibiting thrombus formation (McFadyen et al., 2018). In the study by Kleinschnitz et al. (Kleinschnitz et al., 2007), targeting the early stages of platelet activation (GPIb or GPVI receptors) could protect mice from ischemic brain injury without increasing bleeding complications in an experimental stroke model. Moreover, GPVI deficiencies in humans and mice trigger only a mild bleeding phenotype (Dutting et al., 2012), leading to more interest in targeting GPVI therapeutically.

Heat shock protein 47 (Hsp47) is a collagen-specific molecular chaperone, which plays an essential role in collagen synthesis and/or fibrosis (Khan et al., 2019). In previous studies, Hsp47 was found to be exposed on the surface of human and mice platelets and to strengthen the interaction between platelet and collagen in the formation of thrombi and hemostasis (Kaiser et al., 2009; Sasikumar et al., 2018). Deficiency or inhibition of Hsp47 can inhibit platelet adhesion to collagen, especially the binding of collagen to GPVI receptors (Sasikumar et al., 2018), resulting in attenuating platelet aggregation and thrombus formation. In 2017, Ito et al. discovered a small-molecule inhibitor of the collagen–Hsp47 interaction by screening chemical libraries, called Col003 (5-benzyl-3-nitrosalicylaldehyde). Then, they used nuclear magnetic resonance analysis to confirm that Col003, through competitively binding to the collagen-binding site on Hsp47, inhibited the interaction between them (Ito et al., 2017). However, the effects of Col003 on different platelet function, thrombus formation, and ischemic brain injury remain unknown. Therefore, the present study was conducted to systematically explore these questions and assess whether Col003 is a promising therapeutic agent for ischemic stroke. Some experiments were conducted with aspirin and clopidogrel, which are commonly used antiplatelet drugs in clinical practice, for a comparative analysis. We confirmed that Hsp47 is expressed on the surface of platelets in peripheral blood in normal and MCAO rats and evaluated the antithrombotic efficacy of Col003 at different concentrations. What’s more, we found that Col003 showed low cytotoxicity with normal blood coagulation parameters and bleeding time and not only inhibited collagen-induced platelet activation but also alleviated cerebral ischemic injury in rats.

## Materials and Methods

### Chemicals and Reagents

Col003 (purity ≥ 99%) (HY-124817) was purchased from MCE Chemicals (Shanghai, China) and dissolved in dimethyl sulfoxide (DMSO) throughout the experiments. For *in vivo* experiments, Col003 was reconstituted in 20% (w/v) sulfobutylether-β-cyclodextrin in saline. Aspirin (HY-14654), clopidogrel (HY-15283), arachidonic acid (HY-109590), thrombin (HY-114164), and sulfobutylether-β-cyclodextrin (HY-17031) were purchased from MCE Chemicals (Shanghai, China). Collagen (P/N 385) were purchased from Chrono-Log Crop (Havertown, PA, USA). Fluo-3 acetoxymethyl (AM) and fluorescein isothiocyanate (FITC)-labeled phalloidin were purchased from Sigma-Aldrich (St. Louis, MO, USA). Antibodies against Hsp47 and glyceraldehyde-3-phosphate dehydrogenase (GAPDH) were obtained from Novus Biologicals (Littleton, CO, USA). Antibodies against phospho-Akt (Ser473), phospho-extracellular signal-regulated kinase (ERK)1/2 (Tyr202/204), phospho-protein kinase C (PKC) substrate, total-AKT, and total-ERK1/2 were purchased from Cell Signaling Technology (Danvers, MA, United States). Antibodies against phospho-p38 (Thr180/Tyr182) and phospho–glycogen synthase kinase 3β (GSK3β) (Ser9) were purchased from Proteintech (Wuhan, China). Antibodies against phospho-spleen tyrosine kinase (Syk) (Tyr525), phospholipase C (PLC)γ2 (Tyr1217), total-PLCγ2, total-GSK3β, and total-p38 were purchased from Bioworld (Nanjing, China). Antibodies against phospho-c-Jun N-terminal kinase (JNK) 1/2/3 (Tyr183+Tyr183+Tyr221), total-Syk, and total-JNK1/2/3 were obtained from Boster (Wuhan, China). Phycoerythrin-conjugated anti–P-selectin antibodies (CD62P) and isotype control antibody immunoglobulin G (IgG) were obtained from BioLegend (San Diego, CA, United States).

### Rat Blood Collection and Platelet Preparation

Male Sprague–Dawley rats (280–300 g) were purchased from Animal Experiment Central of Guangxi Medical University and fed in a Specific Pathogen Free (SPF)-level laboratory animal room. All animal experiments were performed according to the guidelines of the Animal Care and Welfare Committee of Guangxi Medical University. Rat whole blood was collected in vacuum tubes containing 3.8% sodium citrate 1:9 (v/v) from the inferior vena cava after rats were anesthetized with chloral hydrate by intraperitoneal injection. Washed platelets were prepared as previously described (Irfan et al., 2018).

### MCAO Model Establishment

The MCAO model used in this study was developed as described previously (Zhang et al., 2020). In brief, after a midline neck incision was made, the external carotid artery (ECA) of the rat was tied, and a silicon-coated nylon suture (4–0) was inserted into the common carotid artery through the internal carotid artery to block the origin of the MCA (approximately 18–22 mm). After a 2-h occlusion period, the nylon suture was gently pulled back to resume blood flow through the MCA. After being awakened from anesthesia, according to a five-point deficit score (Longa et al., 1989), rats with scores in the range of one to three points were regarded as successful models. All rats were anesthetized at 24 h after reperfusion and euthanized for the subsequent experiments.

### Cell Surface Biotin Labeling of Hsp47

Cell surface biotin labeling was performed with the Pierce Cell Surface Protein Biotinylation and Isolation Kit (Thermo Fisher Scientific, Waltham, MA, USA). Washed platelets were first labeled with EZ-Link Sulfo-NHS-SS-Biotin and subsequently lysed with detergent. The labeled proteins were then isolated with NeutrAvidin agarose. The platelet surface presence of Hsp47 was detected by immunoblotting with anti-Hsp47. The immunoblots were reprobed using horseradish peroxidase–conjugated streptavidin antibody to reveal total cell-surface biotinylation. The absence of biotin labeling of intracellular proteins was detected by probing the immunoblots with anti-GAPDH (a negative control for cytosolic proteins).

### FeCl_3_-Induced Carotid Artery Thrombosis Model

Rats were anesthetized by an intraperitoneal injection of 1 ml/100 g of chloral hydrate. Five minutes prior to FeCl_3_-induced vascular injury, rats were injected via the tail vein with 0, 25, 50, or 100 μM of Col003. One of the carotid arteries was exposed and induced by attaching a 3- × 5-mm strip of filter paper saturated with 35% FeCl_3_ solution for 1 minute. Then, a suitable Doppler flow probe was placed on the artery to record the time until the blood flow ceased. The time to occlusion (TTO) was defined as the time from when the FeCl_3_ saturated filter paper was removed to when no blood flow was detectable. The TTO was recorded as 30 min if the blood flow did not cease after 30 min.

### Cell Counting Kit 8 Assay and Lactate Dehydrogenase Assay

Human normal liver L02 cells (5 × 10^3^ cells/well) were seeded in 96-well plates overnight, then incubated with various concentrations of compounds for 24 h. Cell viability was detected with a CCK-8 kit (Dojindo, Kumamoto, Japan). The absorbance at 450 nm was determined using an enzyme standard instrument (Varioskan LUX; Thermo Fisher Scientific, Waltham, MA, USA). Washed platelets were incubated with different concentrations of compounds for 5 minutes. The LDH concentration was detected using an LDH cytotoxicity assay kit (Beyotime Biotechnology, Shanghai, China). The absorbance at 490 nm was recorded using an enzyme standard instrument.

### Coagulation Assessment

After anesthetizing the rats, the required blood samples were collected from the inferior vena cava after 5 minutes of drug injection. Blood samples of different groups were centrifuged at 3,000 g for 10 min to acquire plasma for the measurement of thrombin time, activated partial thromboplastin time, prothrombin time, fibrinogen, and international normalized ratio.

### Tail-Bleeding Assay

Different groups of drugs were infused into the rat circulation via the tail vein 5 minutes prior to performing a tail-bleeding assay. Then, a 2-mm-deep, 8-mm-long incision was created 2–3 cm from the tail root. The blood flow was blotted using filter paper every 15 s for 30 min at the end of the incision until the bleeding stopped and the time was recorded, as described previously (van Ryn et al., 2014).

### Platelet-Aggregation Assay

A total of 250 ul of washed platelets (3 × 10^8^/ml concentration) were incubated with vehicle (0.4% DMSO) or different final concentrations of Col003 (25 or 50 µM) in the presence of CaCl_2_ (1 mM) for 5 minutes at 37°C prior to agonist stimulation. Aggregation was stimulated by agonists collagen (2 μg/ml), arachidonic acid (500 μg/ml), and thrombin (0.1 U/mL) under stirring at 1,200 rpm using an aggregometer (Chrono-Log Corp., Havertown, PA, United States), as previously described (Song et al., 2019).

### Platelet-Adhesion Assay

A total of 250 μl of washed platelet (2 × 10^7^/ml concentration, 1x Tyrodes buffer) containing 1 mM of CaCl_2_ was incubated with Col003 (25 or 50 μM) or vehicle for 5 minutes and subsequently added to the cover slips (coated with 5 μg/ml of collagen) for 1 hour. Finally, the fixed platelets were stained with 1 μg/ml of FITC-labeled phalloidin for 15 min and protected from light, as previously described (Misra et al., 2018). Images from at least three different microscope fields were captured with an Olympus fluorescence microscope (Olympus Corp., Tokyo, Japan) and analyzed with the ImageJ software (United States National Institutes of Health, Bethesda, MD, United States).

### Assessment of the Intracellular Calcium Concentration

Fluo-3 AM was employed to measure the intracellular Ca^2+^ concentrations as described previously (Ya et al., 2021). Washed platelets were incubated with 2 μM of Fluo-3 AM at 37°C for 30 min and protected from light. After 5 minutes of centrifugation at 3,000 g, platelets were resuspended and adjusted the concentration to 1 × 10^8^/ml. Platelets were incubated with different concentrations of Col003 (25 or 50 μM) for 5 minutes, followed by treatment with collagen (2 μg/ml) for an additional 3 minutes at 37°C. The fluorescence intensity was quantified using an enzyme standard instrument with excitation/emission wavelengths of 488 nm/520 nm.

### P-Selectin Expression Analysis by Flow Cytometry

Washed platelets (5 × 10^7^ cells/mL) were stimulated with collagen (2.0 μg/ml) following incubation with vehicle (0.4% DMSO) or Col003 (50 μM) for 5 minutes. The rate of CD62P-positive platelets was detected using flow cytometry analysis as previously reported (Song et al., 2019). A phycoerythrin-labeled, isotype-matched IgG was used as a negative control. Platelets were analyzed by acquiring 10,000 events per sample within the gated population, using a flow cytometer (BD Bioscience, San Diego, CA, United States).

### Measurement of Intracellular Reactive Oxygen Species

The intracellular ROS levels were measured by application of oxidation-sensitive fluorescent probe (DCFH-DA) using a ROS assay kit (Beyotime Biotechnology, Shanghai, China), as previously described (Arthur et al., 2012). Briefly, washed platelets (2.5 × 10^8^/ml) were incubated with 10 μM of DCFH-DA at 37°C for 30 min, pretreated with vehicle (1% DMSO) or Col005 (50 μM), then stimulated with collagen (2.0 μg/ml) followed by recording of the DCF-positive platelets by flow cytometry.

### Immunoblotting

Using a Chrono-Log aggregometer, after incubation with vehicle (0.4% DMSO) or 50 μM of Col003 for 5 minutes at 37°C, washed platelets (3 × 10^8^ cells/mL) were stimulated by collagen (2 μg/ml) for 3 minutes. Aggregated platelets were lysed by RIPA lysis buffer containing protease and phosphatase inhibitor cocktail. Protein samples were separated by 10% sodium dodecyl sulphate–polyacrylamide gel electrophoresis gel and transferred to polyvinylidene difluoride membranes. After incubation with primary antibodies and the corresponding secondary antibodies, the samples were then visualized by enhanced chemiluminescence assay (Beyotime Biotechnology, Shanghai, China). Membranes were stripped and re-probed for β-actin as loading control.

### Neurological Tests

Twenty-four hours after induction of the MCAO model, Bederson’s test and the grip test were performed blindly to evaluate global neurological and motor functions of rats, respectively, as described (Denorme et al., 2020). The Bederson’s scale is defined by: 0, no observable deficit; 1, forelimb flexion; 2, decreased resistance to lateral push (and forelimb flexion) without circling; 3, same behavior as grade 2, with circling. The grip test was carried by placing the rat on a string 50 cm in length and 3 cm in diameter and was rated on the basis of following system: 0, falls off; 1, hangs onto string by two forepaws; 2, as for 1, but attempts to climb onto string; 3, hangs onto string by two forepaws plus one or both hindpaws; 4, hangs onto string by all four paws plus tail wrapped around string; 5, escape.

### 2,3,5-Triphenyltetrazolium Chloride Staining

After the rats were sacrificed, their brains were isolated rapidly and sliced into five coronal 2-mm-thick sections. The sheets were immediately immersed in 2% TTC in phosphate-buffered saline for a 20-min incubation period at 37°C. Sections were subsequently photographed, and the infarction volume was determined by Image-Pro Plus version 6.0 (Media Cybernetics, Rockville, MD, United States). The evaluation of infarct volumes was expressed as a percentage of the contralateral hemisphere.

### Terminal Deoxynucleotidyl Transferase dUTP Nick End Labeling Staining

The TUNEL technique was employed to detect cell apoptosis via an *in situ* cell death detection kit, POD (Roche Holdings, Basel, Switzerland). TUNEL-positive cells (stained with FITC) and all nuclei (stained with DAPI) in the brain tissues were observed and analyzed using an Olympus fluorescence microscope (Olympus Corp., Tokyo, Japan). Results are shown as the percentage of the TUNEL-positive cells.

### Statistical Analysis

Data analysis and presentation were performed using GraphPad Prisim version 6.0 (GraphPad Software, San Diego, CA, United States) and graphed as mean ± standard error of the mean values. Differences between two groups were analyzed using an unpaired two-tailed Student’s *t*-test, or one-way analysis of variance followed by Dunnett’s test. The nonparametric Mann–Whitney *U* test was employed to analyze non–normally distributed data (tail-bleeding assay). *p*-values less or equal than 0.05 were considered statistically significant.

## Results

### Hsp47 Is Present at the Platelet Surface Both in Normal and MCAO Model Rats

**FIGURE 1:**
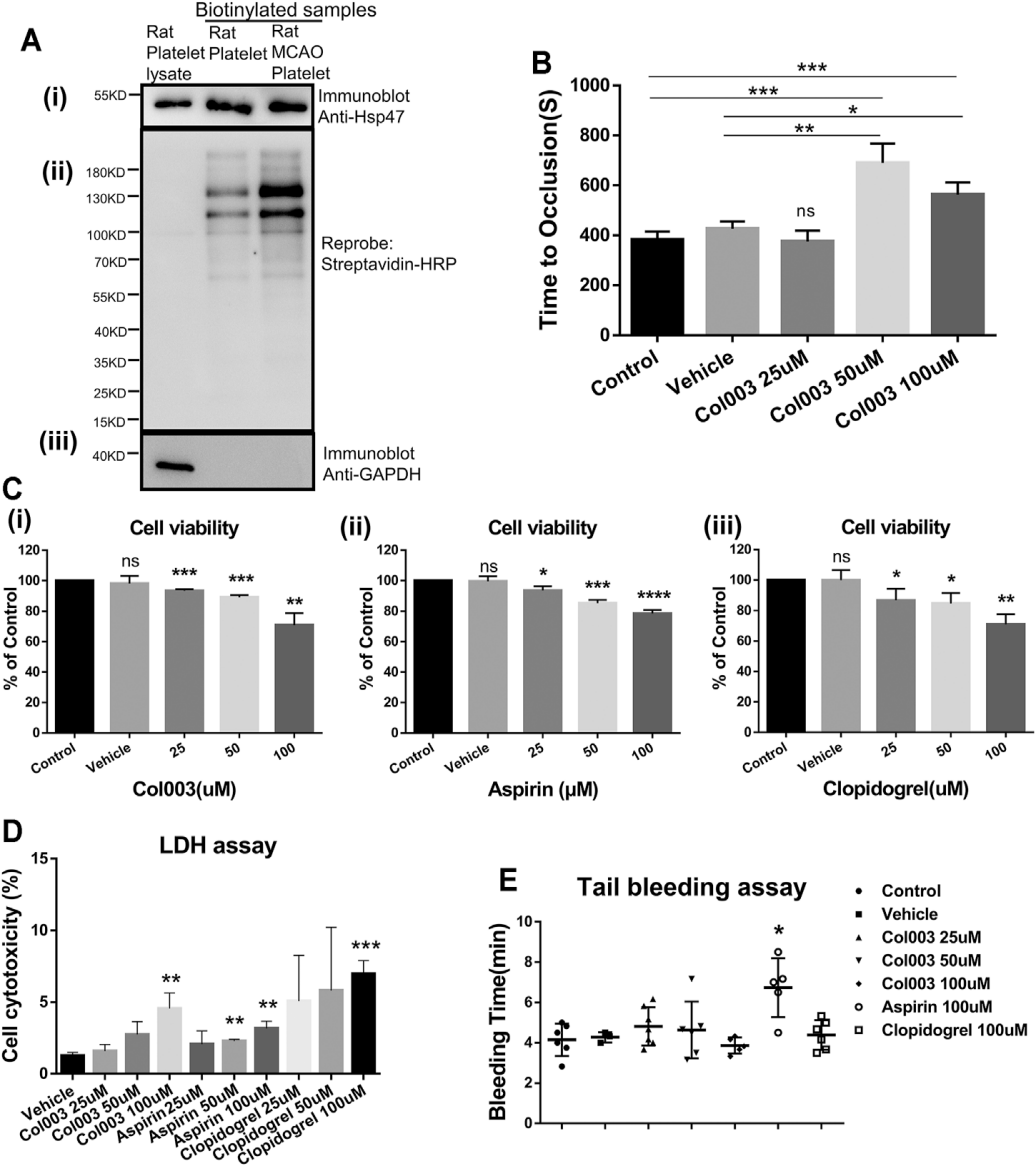
Hsp47 is present at the platelet surface both in normal and MCAO rats, and its inhibitor Col003 attenuates thrombus formation. Col003 had low cytotoxicity on L02 cells and rat platelets without altering the tail-bleeding time in rats. **(Ai)** Biotinylated platelet surface proteins were assessed by immunoblots probed using anti-Hsp47 antibody. **(Aii)** Reprobing the immunoblot with horseradish peroxidase–conjugated streptavidin antibody was conducted to reveal total biotinylation. **(Aiii)** Immunoblots were also reprobed for GAPDH (a cytosolic protein) to confirm that the biotinylation was exclusively of cell surface proteins. **(B)** TTO (s) following FeCl_3_-induced injury of the carotid artery was recorded in different groups. TTO in the five groups were 381.8 ± 14.94 s (Control), 426.3 ± 16.70 s (Vehicle), 375.0 ± 25.03 s (Col003 25uM), 689.3 ± 45.41 s (Col003 50uM), 562.7 ± 28.42 s (Col003 100uM), respectively. *n* = 3–5; *p < 0.05, **p < 0.01 and ***p < 0.001. **(C)** The cell viability of L02 cells after incubation with Col003 (i), aspirin (ii) and clopidogrel (iii) for 24 h was detected by CCK-8 assay. The cell viability of L02 cells in Col003 group were 98.10 ± 2.89% (Vehicle), 93.42 ± 0.53% (25uM), 89.22 ± 0.77% (50uM), 70.90 ± 4.59% (100uM), respectively. The cell viability of L02 cells in aspirin group were 99.53 ± 1.93% (Vehicle), 93.57 ± 1.60% (25uM), 85.39 ± 1.19% (50uM), 78.53 ± 1.31% (100uM), respectively. The cell viability of L02 cells in clopidogrel group were 99.95 ± 3.73% (Vehicle), 86.76 ± 4.34% (25uM), 84.76 ± 3.86% (50uM), 71.00 ± 3.79% (100uM), respectively. *p < 0.05, **p < 0.01, ***p < 0.001, and ****p < 0.0001 (vs control). **(D)** Cytotoxicity in different groups was detected via LDH assay. The cytotoxicity in different groups were 1.27 ± 0.13% (Vehicle), 1.59 ± 0.27% (Col003 25uM), 2.75 ± 0.52% (Col003 50uM), 4.56 ± 0.63% (Col003 100uM), 2.07 ± 0.54% (Aspirin 25uM), 2.29 ± 0.07% (Aspirin 50uM), 3.18 ± 0.29% (Aspirin 100uM), 5.08 ± 1.84% (Clopidogrel 25uM), 5.82 ± 2.55% (Clopidogrel 50uM), 6.97 ± 0.54% (Clopidogrel 100uM), respectively. **p < 0.01 and ***p < 0.001 (vs vehicle). Statistical methods for TTO, cell viability and cytotoxicity using unpaired two-tailed Student’s *t*-test. **(E)** The effect of Col003 on tail-bleeding time in rats. The tail-bleeding time in seven groups were 4.15 ± 0.33 min (Control), 4.28 ± 0.15 min (Vehicle), 4.81 ± 0.36 min (Col003 25uM), 4.64 ± 0.56 min (Cl003 50uM), 3.87 ± 0.18 min (Col003 100uM), 6.73 ± 0.65 min (Aspirin 100uM), 4.39 ± 0.30 min (Clopidogrel 100uM), respectively. Statistical analysis was performed with the nonparametric Mann–Whitney *U* test (*n* = 3–7; *p < 0.05, vs control).

Immunoblotting analysis of isolated biotinylated proteins revealed the expression of Hsp47 on the platelet surfaces (Figure 1Ai). The protein bands of total rat platelet lysate were absent, confirming that the platelet surface biotinylation of proteins was successful (Figure 1Aii). Meanwhile, Figure 1 (Aii) shows the differential biotin labeling between normal and MCAO rat platelets, consistent with the acknowledged upregulation of proteins on the cell surface following platelet activation in the MCAO model. GAPDH was used as a negative control to confirm platelet integrity during cell surface biotinylation (Figure 1Aiii).

### Col003 Suppresses FeCl_3_-Induced Carotid Artery Thrombosis in Rats

The average TTO after FeCl_3_ exposure was 381.8 ± 14.94 s and 426.3 ± 16.70 s in the control group and vehicle group, respectively, and no statistical difference was observed when the 25-μM of Col003 injection group (375.0 ± 25.03 s) was compared with either (Figure 1B). However, the 50-μM of Col003 injection group (689.3 ± 45.41 s) and 100-μM of Col003 injection group (562.7 ± 28.42 s) exhibited significantly prolonged TTO values after FeCl_3_ injury (Figure 1B).

### Cytotoxicity of Col003 on Human Normal Liver L02 Cells and Rat Platelets

As shown in Figs. 1Ci, 1Cii, and 1Ciii, Col003, aspirin, and clopidogrel reduced the cell viability of L02 cells in a dose-dependent manner. The results showed that the degree of cytotoxicity was similar among Col003, aspirin, and clopidogrel. Both 25-μM and 50-μM doses of Col003 had low cytotoxicity against L02 cells (cell viability >89.22% of control). LDH assay results revealed an upward trend of the cytotoxicity of Col003 with increasing concentrations, but no cytotoxicity was observed at 25 or 50 μM (Figure 1D). Although 50 and 100 μM aspirin and 100 μM clopidogrel showed some degree of cytotoxicity to rat platelets, the cytotoxicity of all agents in this study was less than 10% (Figure 1D).

### Effects of Col003 on Plasma Coagulation Parameters and Bleeding Time in Rats

No change in plasma coagulation parameters was observed in correlation with the various concentrations of Col003 treatment nor with 100 μM of aspirin or 100 μM of clopidogrel (Table 1). Three different concentrations of Col003 and 100 μM of clopidogrel did not affect the tail-bleeding time in rats compared with in control or vehicle-treated rats (Figure 1E). Under the same conditions, aspirin prolonged the tail-bleeding time by up to 6.73 ± 0.65 min, and its effect was statistically significant relative to the control group result (4.15 ± 0.33 min) (*p* < 0.05).

**TABLE 1:** Effect of Col003, aspirin, and clopidogrel on plasma coagulation parameters *in vivo* (mean ± SD, n = 3–4).

Group	PT (s)	TT (s)	APTT (s)	FIB (g/L)	INR
Control	9.27 ± 0.15	39.43 ± 1.00	16.77 ± 0.78	2.15 ± 0.02	0.78 ± 0.01
Vehicle	8.5 ± 0.17	35.27 ± 4.27	26.27 ± 4.63*	2.15 ± 0.10	0.72 ± 0.01
Col003 25 μM	9.3 ± 0.30	41.18 ± 3.02	20.78 ± 1.02	2.05 ± 0.31	0.79 ± 0.03
Col003 50 μM	9.47 ± 0.33	42.87 ± 0.46	19.43 ± 1.53	1.77 ± 0.17	0.80 ± 0.03
Col003 100 μM	8.53 ± 0.43	33.40 ± 5.62	18.60 ± 0.51	2.60 ± 0.15	0.72 ± 0.04
Aspirin 100 μM	9.33 ± 0.25	36.10 ± 0.90	18.58 ± 0.73	2.24 ± 0.16	0.79 ± 0.02
Clopidogrel 100 μM	9.27 ± 0.23	38.40 ± 0.61	17.53 ± 0.96	2.18 ± 0.13	0.78 ± 0.02

Abbreviations APTT, activated partial thromboplastin time; FIB, fibrinogen; INR, international normalized ratio; PT, prothrombin time; TT, thrombin time.

Statistical analysis was performed with one-way analysis of variance followed by Dunnett’s test (Compared with the control group: *p < 0.05).

### Effect of Col003 on Rat Platelet Aggregation Induced by Various Agonists

**FIGURE 2:**
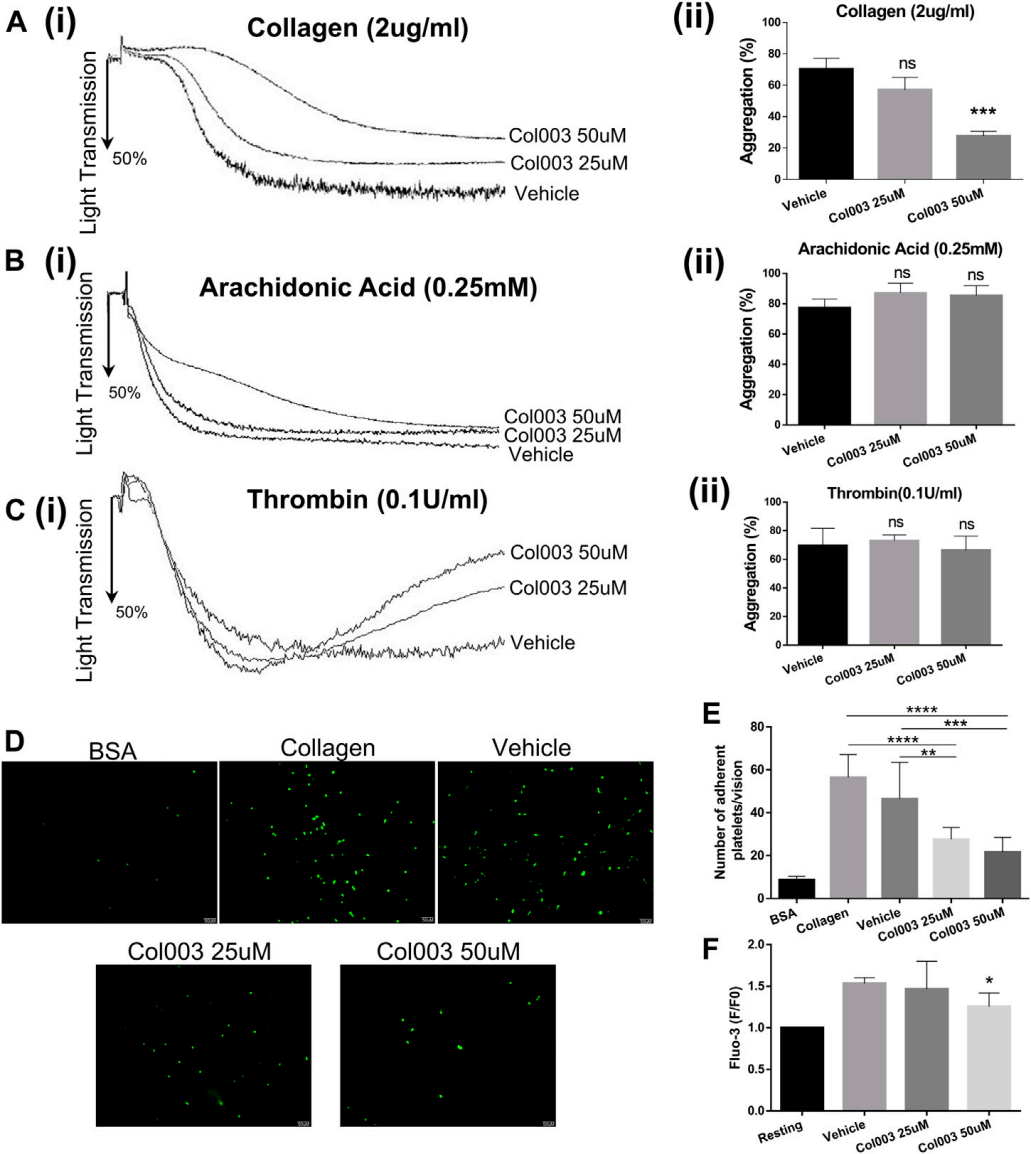
Effects of Col003 on platelet aggregation induced by various agonists and platelet adhesion and Ca^2+^ intra-platelet calcium flux induced by collagen. Washed rat platelets (3 × 10^8^/ml) were pre-incubated 5 minutes with the indicated concentrations of Col003 or vehicle followed by the addition of collagen **(Ai)**, arachidonic acid **(Bi)**, or thrombin **(Ci)**. The aggregation rates (%) were quantified (Aii, Bii, Cii; ***p < 0.001, vs vehicle). The aggregation rates (%) in collagen-induced group were 70.33 ± 3.93% (Vehicle), 57.00 ± 4.58% (Col003 25uM), 27.67 ± 1.76% (Col003 50uM), respectively. The aggregation rates (%) in arachidonic acid-induced group were 77.33 ± 3.28% (Vehicle), 87.00 ± 3.79% (Col003 25uM), 85.33 ± 3.84% (Col003 50uM), respectively. The aggregation rates (%) in thrombin-induced group were 69.67 ± 6.89% (Vehicle), 73.00 ± 2.31% (Col003 25uM), 66.33 ± 5.70% (Col003 50uM), respectively. **(D)** Representative images of platelet adhesion on collagen-coated round coverslips. **(E)** The number of adherent platelets per vision was quantified. The adherence number of platelets in the five groups were 8.68 ± 0.58 platelets/vision (BSA), 56.44 ± 3.55 platelets/vision (Collagen), 46.44 ± 5.69 platelets/vision (Vehicle), 27.56 ± 1.88 platelets/vision (Col003 25uM), 21.67 ± 2.30 platelets/vision (Col003 50uM), respectively. **p < 0.01, ***p < 0.001, and ****p < 0.0001. **(F)** The intracellular Ca^2+^ concentration was measured by labeled Fluo-3 AM, and the Fluo-3 fluorescence of platelets was presented normalized to the fluorescence of resting platelets. The [Ca2+]i levels in the three groups were 1.53- ± 0.04-fold of resting (Vehicle), 1.47- ± 0.17-fold of resting (Col003 25uM), 1.26- ± 0.08-fold of resting (Col003 50uM), respectively. *p < 0.05 (vs vehicle). Statistical methods for the above experiments using unpaired two-tailed Student’s *t*-test.

Considering the cytotoxicity of Col003, both 25 and 50 μM concentrations were chosen for further platelet function study. Adopting 2 μg/ml of collagen as an agonist, 25 μM of Col003 tended to decrease the aggregation rates, but this finding did not reach statistical significance compared with vehicle (57.00 ± 4.58% vs 70.33 ± 3.93%; *p* = 0.0918; Figure 2A). At the same collagen concentrations, 50 μM of Col003 notably reduced the aggregation rates in rat platelets (27.67 ± 1.76% vs 70.33 ± 3.93%; *p* < 0.001; Fig. 2Aii). Col003 showed no inhibitory effect on arachidonic acid- or thrombin-induced platelet aggregation (Figures 2B,C).

### Effect of Col003 on Rat Platelet Adhesion to Collagen

As shown in Figure 2D, platelet adhesion to collagen was significantly lower in rats treated with 25 or 50 μM of Col003 compared to control and vehicle rats. Statistical results are presented in Figure 2E (56.44 ± 3.55 platelets/vision at control, 46.44 ± 5.69 platelets/vision at vehicle, 27.56 ± 1.88 platelets/vision at 25 μM of Col003 and 21.67 ± 2.30 platelets/vision at 50 μM of Col003), and all differences were statistically significant.

### Effect of Col003 on Collagen-Induced Ca^2+^ Intra-platelet Calcium Flux

Study results showed that 50 μM of Col003 significantly decreased the collagen-induced mobilization of intra-platelet [Ca^**2+**^]_i_. The [Ca^**2+**^]_i_ levels were reduced to 1.47- ± 0.17-fold and 1.26- ± 0.08-fold of resting at concentrations of 25 and 50 μM, respectively, when compared with 1.53- ± 0.04-fold of resting in the vehicle group (Figure 2F).

### Effect of Col003 on Collagen-Induced Expression of P-Selectin

**FIGURE 3:**
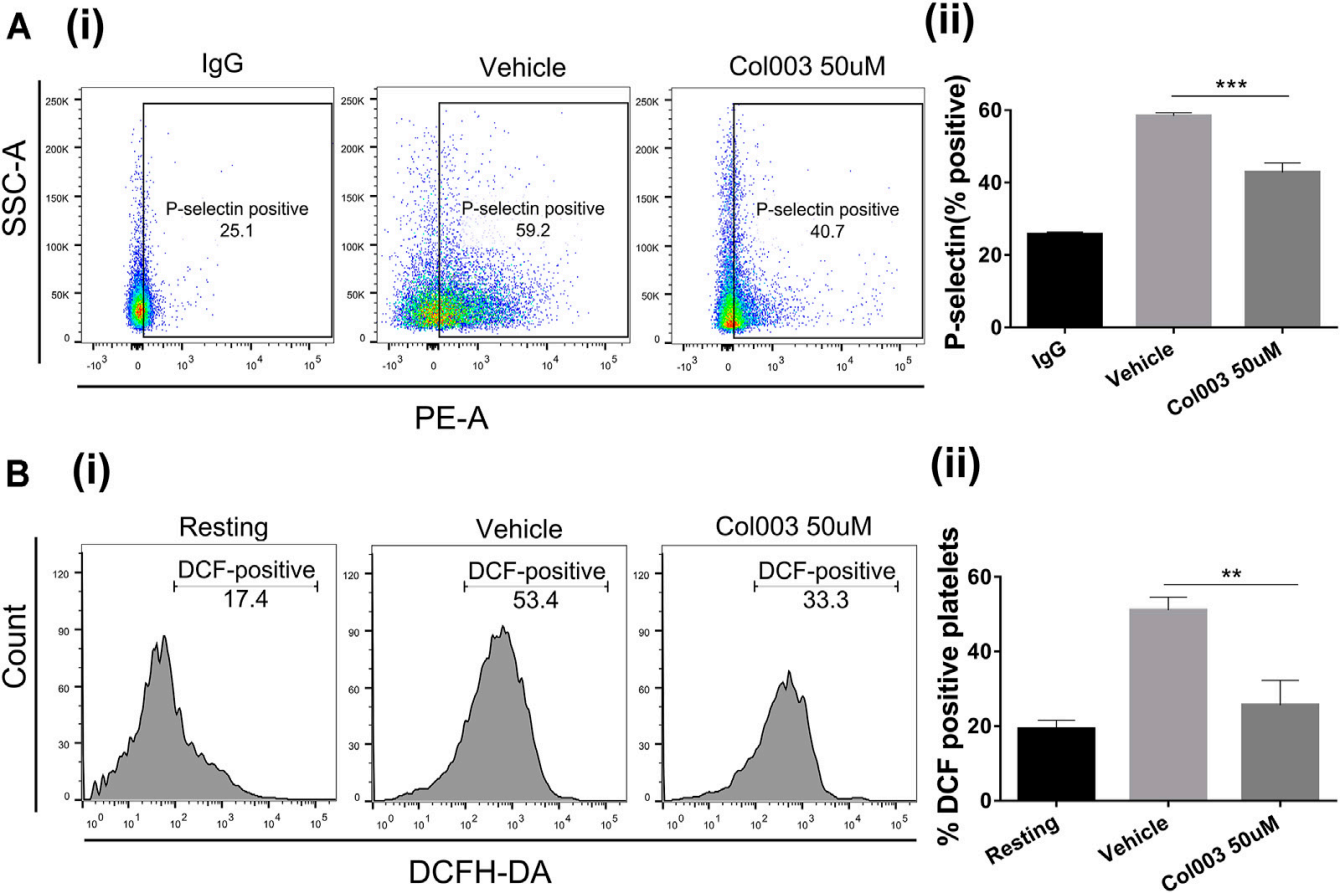
Effects of Col003 on P-selectin expression and ROS generation induced by collagen in rat platelets. **(Ai)** Representative scatterplots of P-selectin expression in IgG (negative control), vehicle, and Col003-treated platelets **(Aii)** The percentage of P-selectin–positive platelets were quantified. The %P-selectin–positive platelets in three different groups were 25.73 ± 0.35% (IgG), 58.43 ± 0.50% (Vehicle), 42.83 ± 1.49% (Col003), respectively. **(Bi)** Flow cytometry of DCHF-DA–loaded platelets in washed platelets treated with either vehicle or Col003 showing collagen-induced ROS generation. **(Bii)** The percentage of DCF-positive platelets was quantified. The %DCF-positive events in different three groups were 19.30 ± 1.31% (Resting), 51.08 ± 1.74% (Vehicle), 25.60 ± 3.85% (Col003 50uM), respectively. Statistical analysis was performed with unpaired two-tailed Student’s *t*-test (**p < 0.01 and ***p < 0.001).

The results of platelet aggregation, adhesion [Ca^**2+**^]_i_ mobilization *ex vivo*, and thrombus formation *in vivo* revealed that 50 μM was the optimal effective concentration of Col003, with low cytotoxicity. Thus, 50 μM of Col003 was chosen to assess its effect on platelet activation by flow cytometry. Representative scatterplots demonstrate the difference in P-selectin expression between IgG (negative control), vehicle, and Col003-treated platelets (Figure 3Ai). The results showed that Col003 decreased the expression of P-selectin on platelets stimulated by collagen compared to vehicle (42.83 ± 1.49% vs 58.43 ± 0.50%; *p* < 0.001; Figure 3Aii).

### Effect of Col003 on Collagen-Induced Endogenous Generation of ROS

Figure 3Bi shows the effect of Col003 (50 µM) on the production of platelet ROS induced by collagen, presented as %DCF-positive events. A significant decrease in ROS production was observed in platelets treated with Col003 compared to platelets treated with vehicle (25.60 ± 3.85% vs 51.08 ± 1.74%; *p* < 0.01; Figure 3Bii).

### Effect of Col003 on Collagen-Induced Signal Transduction

**FIGURE 4:**
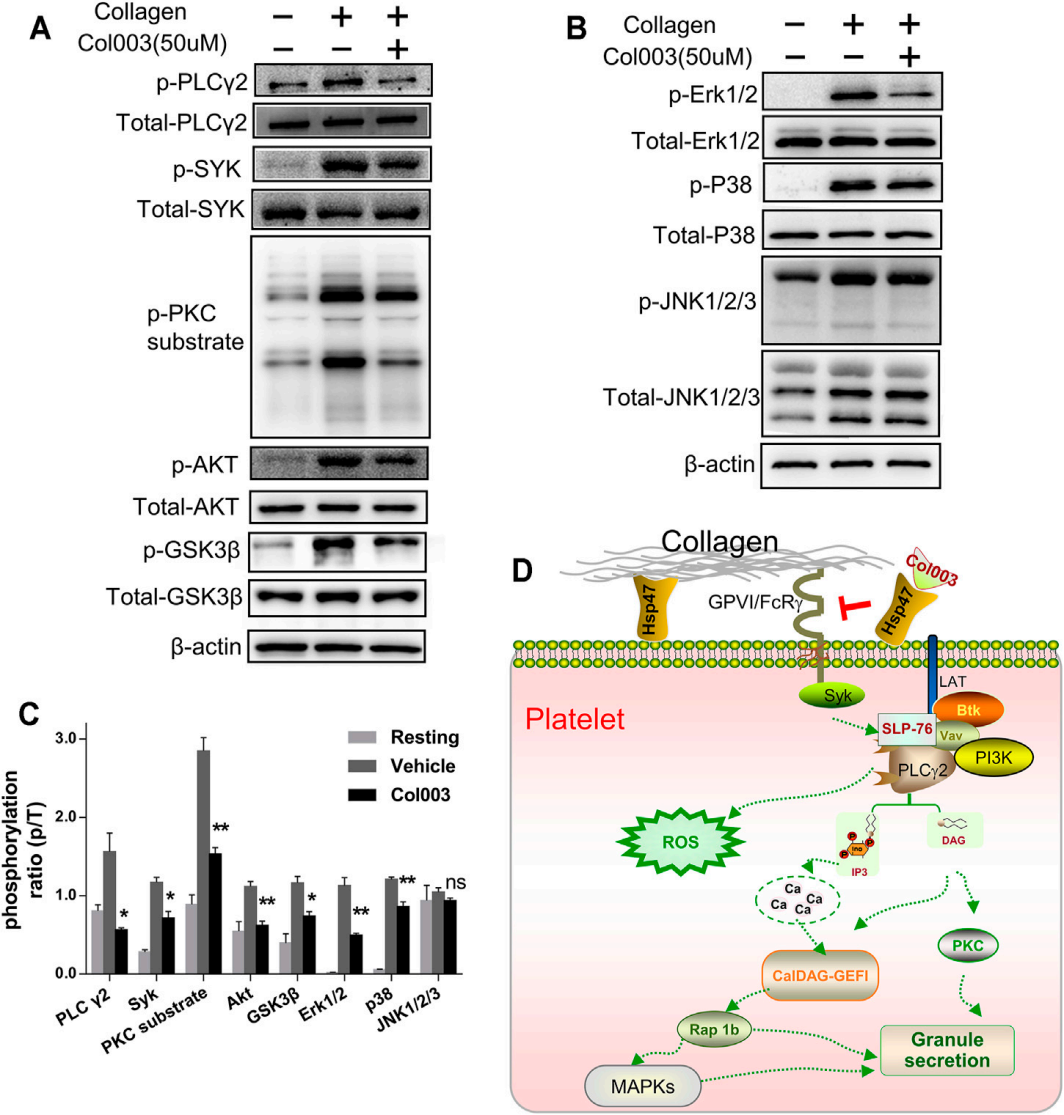
Col003 inhibits collagen-induced phosphorylation of signaling molecules. **(A)** Col003 inhibits phosphorylation of the GPVI signaling pathway induced by collagen. **(B)** Col003 attenuated collagen-activated platelet p38 and ERK2 phosphorylation. **(C)** The values shown in the bar graphs are representative of three independent experiments. Statistical analysis was performed with unpaired two-tailed Student’s *t*-test. *p < 0.05 and **p < 0.01 (vs vehicle). **(D)** Schematic representation of the signaling pathways may be involved in the antiplatelet effect of Col003 induced by collagen.

In this study, Col003 had a strong inhibitory effect on collagen-induced platelet activation. Thus, we analyzed the phosphorylation of several proteins downstream of the GPVI signaling pathway, which is a primary receptor for collagen. We found that the phosphorylation of Syk, PLCγ2, and PKC substrate in the collagen-induced GPVI signaling pathway was significantly inhibited by treatment with 50 μM of Col003 (Figure 4A). Given the relevance of Phosphatidylinositol 3-kinase (PI3K) in GPVI-mediated platelet activation, we investigated the phosphorylation of effector molecules downstream of PI3K signaling, including AKT and GSK3β. The results suggested that, after pre-incubation with Col003, AKT and GSK3β phosphorylation were suppressed in collagen-activated platelets (Figure 4A). These findings were all statistically significant (Figure 4C) and demonstrated that Col003 might negatively regulate platelet GPVI signaling.

Mitogen-activated protein kinase (MAPK) family members have been identified in platelets and activated by various agonists in previous studies (Adam et al., 2008). As shown in Figure 4B, collagen stimulation led to the activation of all three MAPKs (Erk1/2, p38, and JNK1/2/3) in platelets, and only the phosphorylation of JNK1/2/3 in 50 μM of Col003-treated rat platelets was not changed (Figure 4C). Taken together, Col003 might inhibit platelet activation via impairing GPVI and MAPK signaling events (Figure 4D).

### Col003 Treatment Protects Rats From Cerebral Ischemia–Reperfusion Injury and Improves Neurological Functional Outcome

**FIGURE 5:**
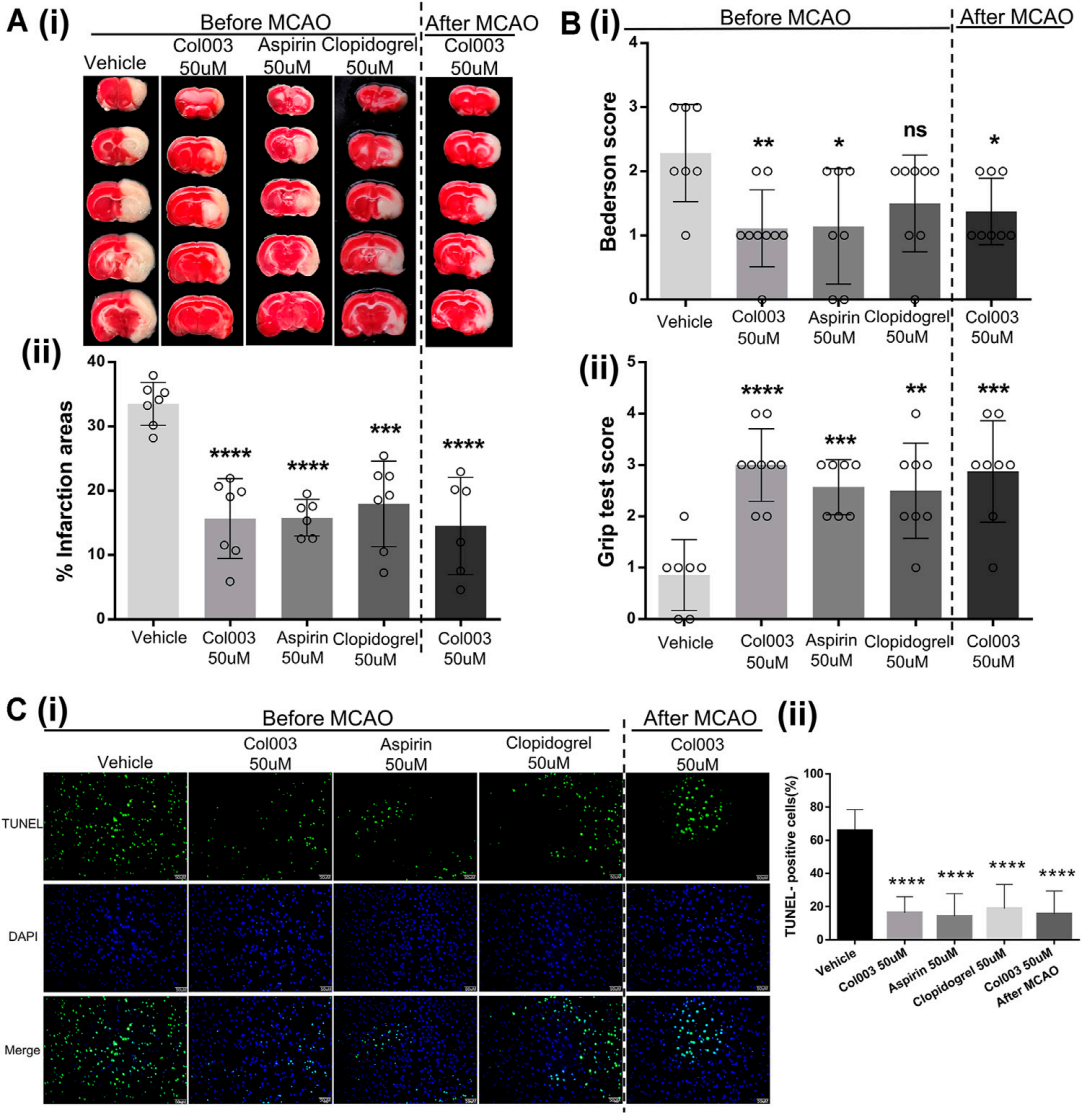
The cerebral infarct volume and outcome of rats after MCAO. **(Ai)** Representative brain sections stained with TTC. Healthy tissue appeared red, whereas infarct areas stained white. **(Aii)** Brain infarct volumes of different groups were quantified by planimetric analysis. The infarction areas (%) in the five groups were 33.52 ± 1.26% (Vehicle), 15.66 ± 2.34% (Col003 50uM before), 15.81 ± 1.17% (Aspirin 50uM before), 17.95 ± 2.52% (Clopidogrel 50uM before), 14.53 ± 3.09% (Col003 50uM after), respectively. **(Bi)** Bederson’s test was used to access neurological outcome. The Bederson’s score in the five groups were 2.29 ± 0.29 points (Vehicle), 1.11 ± 0.20 points (Col003 50uM before), 1.14 ± 0.34 points (Aspirin 50uM before), 1.50 ± 0.27 points (Clopidogrel 50uM before), 1.38 ± 0.18 points (Col003 50uM after), respectively. **(Bii)** Grip strength test was used to examine the motor function. The grip score in the five groups were 0.86 ± 0.26 points (Vehicle), 3.00 ± 0.24 points (Col003 50uM before), 2.57 ± 0.20 points (Aspirin 50uM before), 2.50 ± 0.33 points (Clopidogrel 50uM before), 2.88 ± 0.35 points (Col003 50uM after), respectively. *n* = 7–9; *p < 0.05, **p < 0.01, ***p < 0.001, and ****p < 0.0001 (vs vehicle group). **(Ci)** Representative micrographs of TUNEL staining. **(Cii)** Quantitation of TUNEL-positive neurons. The TUNEL-positive cells (%) in the five groups were 65.80 ± 3.51% (Vehicle), 16.39 ± 2.76% (Col003 50uM before), 14.21 ± 4.32% (Aspirin 50uM before), 18.90 ± 4.59% (Clopidogrel 50uM before), 15.68 ± 4.32% (Col003 50uM after), respectively. *n* = 3; ****p < 0.0001 (vs vehicle group). Statistical methods for the above experiments using unpaired two-tailed Student’s *t*-test.

To further verify the effects of Col003 on brain ischemia, the optimal concentration (50 μM) of Col003 was intravenously injected into the tails of the rats 5 minutes before MCAO, and the same experiment was conducted using aspirin and clopidogrel. The results showed that infarct volumes of the cerebral cortex were reduced dramatically in the 50-μM Col003 group compared to in the vehicle group (15.66 ± 2.34% vs 33.52 ± 1.26%; *p* < 0.0001; Figure 5A). Similarly, treatment with aspirin and clopidogrel significantly diminished the infarct volume (15.81 ± 1.17% and 17.95 ± 2.52%, respectively; *p* < 0.0001 and *p* < 0.001; Figure 5A). Consistent with the reduction in infarct size after Col003 treatment, the global neurological function (assessed by Bederson’s score) and motor function (measured by the grip test) were significantly better than those in the vehicle group (Figure 5B) (Bederson’s score, 2.29 ± 0.29 points vs 1.11 ± 0.20 points; *p* < 0.01; grip test, 0.86 ± 0.26 points vs 3.00 ± 0.24 points; *p* < 0.0001). To test whether Col003 is also beneficial in the acute phase after focal cerebral ischemia, Col003 was applied 5 minutes after the induction of MCAO. The protective effect observed with Col003 infusions before MCAO model creation was observed similarly with Col003 infusions after MCAO model creation, which revealed that this therapeutic approach was as efficacious as it was prophylactic. Taken together, these results indicate that the platelet surface protein Hsp47 may play an important role in stroke development after MCAO, and its inhibitor Col003 may be helpful for ischemic stroke treatment.

Compared to in the vehicle group, fewer apoptotic neurons were observed in the aspirin infusion group, clopidogrel infusion group, Col003 infusion before MCAO model creation group, and Col003 infusion after MCAO model creation group (Figure 5C). These findings indicate that Col003 reduced apoptotic neuronal death after MCAO model creation in rats, and this effect was also apparent with aspirin and clopidogrel.

## Discussion

Hsp47 is a 47-kDa collagen-binding glycoprotein localized in the endoplasmic reticulum, which has been frequently reported in association with collagen-related disorders, such as fibrosis (Ito and Nagata, 2019). On the other hand, some studies have shown that Hsp47 is expressed on the surface of human and mice platelets and promotes the adhesion of platelets to collagen (Kaiser et al., 2009; Sasikumar et al., 2018). Our study shows for the first time that Hsp47 is also expressed on the surface of rat platelets. An FeCl_3_-induced arterial injury model is commonly used to assess antithrombotic effect and is believed to stimulate the platelet responses that underlie pathogenic arterial thrombus formation in humans (Schmaier et al., 2011). Our results showed that a suitable concentration of Col003 significantly prolonged the TTO, which suggests that the inhibition of Hsp47 could produce an antithrombotic effect.

Current antiplatelet drugs all carry an inherent risk of bleeding, leading to a benefit on stroke progression and recurrence that is often offset by a significant increase in the odds of bleeding (Mackman et al., 2020). The combination of a bleeding model and coagulation parameter detection can help in assessing side effects of Col003. Following the injection of various concentrations of Col003, rats did not exhibit prolonged tail bleeding time (even shorter than that of aspirin) or change the coagulation parameters, similar to as seen with an injection of clopidogrel. Meanwhile, the low toxicity of Col003 to rat platelets and human liver cells was similar to that of both aspirin and clopidogrel, especially at doses of 25 and 50 μM. Taken together, Col003 may be a safe antiplatelet drug with few side effects.

In the present study, we found that Col003 possesses a strong suppression effect on collagen-induced rat platelet aggregation, consistent with previously reported data in mice (Sasikumar et al., 2018). However, arachidonic acid–or thrombin-induced aggregation could not be inhibited by Col003, which showed that Col003 has potential specificity for collagen receptors. This supports the idea that Hsp47 might be one of the collagen receptors on the platelet surface able to trigger platelet activation during thrombosis. Platelet adhesion to collagen and Ca^2+^ mobilization induced by different agonists have been reported as essential steps in the process of investigating hemostasis and thrombosis (Wei and Harper, 2019). Our present study showed that platelet adhesion to collagen and the collagen-induced platelet intracellular calcium concentration were attenuated by Col003 treatment, which further confirmed its good antiplatelet effect. The above experimental results suggest the optimal effective therapeutic concentration of Col003 is 50 μM.

P-selectin is a component in the platelet α-granules and is expressed on the platelet surface membrane upon activation (Leytin et al., 2000). The expression of P-selectin on the cell membrane can reflect the release of platelet α-granules (Song et al., 2019) and has been widely used to characterize platelet activation in various experimental and clinical conditions. ROS are natural by-products of aerobic metabolism and play an important role in regulating the response of platelets to collagen and collagen-mediated thrombosis (Qiao et al., 2018). Upon platelet activation, platelet-derived ROS, in turn, boost further ROS generation and consequent platelet activation (Masselli et al., 2020). In our study, the expression of P-selectin and intracellular ROS levels were decreased by Col003, suggesting that Col003 can suppress platelet release function and reduce collagen-induced platelet activation.

GPVI is platelet-specific and has now been recognized as a major platelet receptor for collagen, crucial for both platelet adhesion and thrombus formation. Because of the favorable role of Col003 on the inhibition of collagen-induced activation, we focused on GPVI signaling to study the possible mechanisms of Col003 on antiplatelet function. Activation of the GPVI collagen receptor results in Syk activation, followed by the recruitment of PI3K and PLCγ2; the latter then liberates Inositol trisphosphate (IP3) and Diacylglycerol (DAG) for subsequent PKC activation and [Ca^2+^]_i_ mobilization (Endale et al., 2012). PI3K has been extensively studied in platelets and is believed to play an important role downstream of the GPVI receptor (Martin et al., 2010). Our finding suggests that Col003 can inhibit the Syk–PLCγ2–PKC and PI3K–Akt–GSK3β signaling pathway. Since GPVI is strongly involved in the pathogenesis of arterial thrombosis without a great impact on normal hemostasis (Borst and Gawaz, 2020), GPVI moved into focus during work on novel potential antiplatelet candidates for antithrombotic therapy. Our study confirmed that Col003 inhibits platelet activation and thrombus formation via suppressing the GPVI signaling pathway, which suggests that Col003 may be a promising antiplatelet agent.

According to the literature, the ERK2 activation required for platelet adhesion is dependent on the interaction of GPIb with von Willebrand factor, while p38 was reported to be involved in collagen-induced platelet adhesion and spreading (Mazharian et al., 2005). Our observations in the present study indicate that Col003 markedly inhibits collagen-induced ERK2 and p38, suggesting that the modulation of the MAPK signaling pathway may be involved in Col003 antiplatelet activity. Overall, after competitively binding to the collagen-binding site on Hsp47, Col003 inhibits collagen-stimulated platelet function through the regulation of signaling downstream of the collagen receptor GPVI (Figure 4D).

MCAO is the most widely used animal model of stroke, often used to assess the effects of some new treatments for ischemic stroke. Interestingly, in our study, reduced stroke volumes after Col003 injection were accompanied by a significant reduction in neurological deficits, even when Col003 was injected with a delay of 5 minutes after the induction of MCAO. This indicates its potential suitability for clinical application in the acute phase of ischemic stroke in humans, in whom treatment options are very limited. It is well-known that apoptosis during cerebral ischemic–reperfusion injury plays an important role in the process of neuronal death, which can induce brain damage and exist for an extended period (Tang et al., 2020). The reduction in neuronal apoptosis caused by Col003 injection in the MCAO model further verifies its benefit on ischemic injury. Overall, our study indicates the existence of a protective role for Col003 in regulating brain injury induced by ischemic stroke.

In conclusion, we first confirmed the expression of Hsp47 on rat platelets. The Hsp47 inhibitor Col003 can suppress collagen-induced aggregation, adhesion [Ca^2+^]_i_ mobilization, P-selectin expression, ROS production *in vitro*, and thrombus formation *in vivo* without affecting coagulation parameters and the bleeding time. The underlying mechanism is accomplished via inhibiting Syk–PLCγ2–PKC/MAPK and PI3K–Akt–GSK3β activation downstream of the GPVI signaling cascades. Meanwhile, Col003 can protect rats from cerebral ischemic injury in an MCAO model. At present, few studies have investigated the effects of Hsp47 and its inhibitor on platelet activation, let alone the effects on cerebrovascular diseases. Thus, our study might offer a new antiplatelet agent and strategy to treat ischemic stroke.

## Data Availability

The raw data supporting the conclusions of this article will be made available by the authors, without undue reservation.
